# Assessment of right and left ventricular trabeculation in a reference collective: gender and age dependency of myocardial trabeculation

**DOI:** 10.1186/1532-429X-13-S1-P273

**Published:** 2011-02-02

**Authors:** Astrid Burger, Stephanie Lehrke, Dirk Lossnitzer, Grigorios Korosoglou, Evangelos Giannitsis, Hugo A Katus, Henning Steen

**Affiliations:** 1 University Heidelberg, Heidelberg, Germany

## Introduction

Left ventricular non-compaction (LVNC) cardiomyopathy is characterized by a thin, compacted epimyocardial and a thick non-compacted, trabeculated endomyocardial layer. High-resolution cardiac magnetic resonance imaging (CMR) has been successfully used to distinguish myocardial trabeculation within the LV cavity. Usually, a compact-to-non-compact ratio of 2.3 on MRI is regarded pathological.

Unfortunately, this one-dimensional measure is not well standardized and observer-dependent. Moreover, only scarce data exists on age and gender dependency of myocardial trabeculation in normal volunteers

## Purpose

We present a novel multi-slice measurement approach for myocardial trabeculations and sought to investigate age and gender dependencies on LV and RV myocardial trabeculations.

## Methods

In 120 male/female healthy volunteers divided into three age groups (1=20-35ys;2=36-50ys.;3=>51ys) a vector-ECG gated multi-slice short axis standard cine SSFP-sequence was used. Trabeculation volume was measured by drawing contours between compacted and non-compacted myocardium (outer solid line) as well as between edges of the trabeculation net and normal end-diastolic LV volume (inner dashed line) and normalized to body mass index (BMI). Papillary muscle were excluded. Data was compared using ANOVA (p<0.05 significant).

## Results

**Figure 1:**
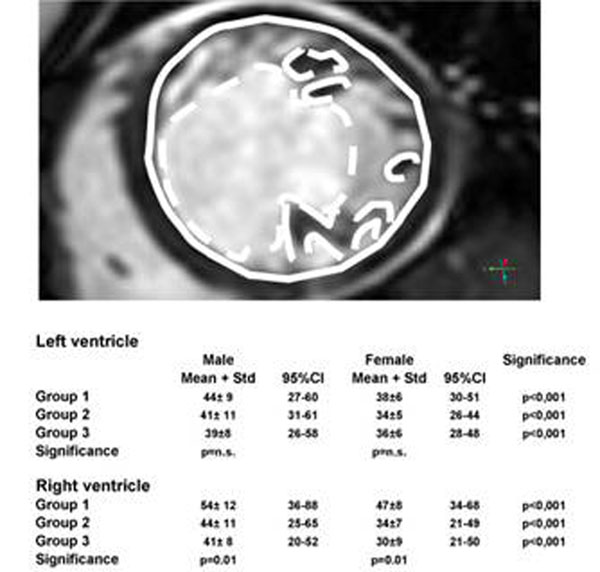


Results are presented in figure 1. For myocardial trabeculation in the LV, there was no age dependency in male and female volunteers but strong gender dependency for all age groups (all p<0.001), whereas male volunteers showed higher volumes in all groups. In contrast, for RV myocardial trabeculation, there was both strong age dependency in male and female volunteers (p=0.01) and strong gender dependency for all age groups (all p<0.001**).**

## Conclusions

Interestingly, myocardial trabeculation is different between RV and LV. Though the LV trabeculation is independent from age for women and men, there is a significant decrease of trabeculation volume in the RV. Male volunteers always reveal higher trabeculation volumes for all age groups in both ventricles

